# The deployment of semiochemical-based pest management tools for woody plants in managed residential and commercial landscapes: current status, challenges, and future directions

**DOI:** 10.1093/jisesa/ieag109

**Published:** 2026-09-17

**Authors:** Chad M Rigsby, Christopher B Riley, Jeremy D Slone, Margaret C Marshall, Amber R Stiller, Jessie J Ward, Kevin D Chase

**Affiliations:** The R.A. Bartlett Tree Research Laboratories and Arboretum, Charlotte, NC, USA; Center for Tree Science, The Morton Arboretum, Lisle, IL, USA; The R.A. Bartlett Tree Research Laboratories and Arboretum, Charlotte, NC, USA; Casey Trees Farm, Berryville, VA, USA; The R.A. Bartlett Tree Research Laboratories and Arboretum, Charlotte, NC, USA; The R.A. Bartlett Tree Research Laboratories and Arboretum, Charlotte, NC, USA; Center for Tree Science, The Morton Arboretum, Lisle, IL, USA; The R.A. Bartlett Tree Research Laboratories and Arboretum, Charlotte, NC, USA; The R.A. Bartlett Tree Research Laboratories and Arboretum, Charlotte, NC, USA; Casey Trees Farm, Berryville, VA, USA; The R.A. Bartlett Tree Research Laboratories and Arboretum, Charlotte, NC, USA

**Keywords:** pheromones, kairomones, ornamentals, monitoring, trapping

## Abstract

Managed residential and commercial landscapes are primarily managed for aesthetic purposes or to provide social benefits (e.g. shade). These landscapes, and the woody plants that comprise them, exist in environments that pose unique challenges for the management of pests, such as the discontinuous nature and patchiness of habitat and hosts, abiotic conditions that result in plant stress, and the existence of ecologically arbitrary but legally meaningful boundaries. Due to challenges like these and other factors, plant health care managers have heavily relied upon traditional pesticides for decades, but with the legal and cultural dynamics surrounding traditional pesticides changing in many parts of the world, novel strategies need development. While semiochemical-based pest management strategies offer an enticing alternative (or supplement) to traditional pesticides and have been in use for many years in these landscapes, they have primarily been developed for use in very different environments, such as forestry plantations, orchards, and natural forests, that have very different biological and spatial dynamics. In this paper, we discuss the factors that make semiochemical pest management in residential and commercial landscapes different from others, case studies of what semiochemical strategies have worked, and what semiochemical research should be conducted going forward that allows the green industry to adopt semiochemical tools into an integrated pest management program.

## Introduction

The sudden availability of inexpensive, effective synthetic chemical insecticides in the 1940s resulted in heavy reliance on these new products, specifically to suppress pest outbreaks (Flint and van den Bosch 1981, Nielson 1983, Wu et al. 1991, Kogan 1998). The impacts of widespread and relatively non-targeted pesticide use became increasingly apparent within a few decades, including pesticide resistance, pest resurgence, and secondary pest outbreaks (Kogan 1998). In response, research began to center on the integration of a variety of strategies for insect management, and the framework for the integrated pest management (IPM) approach was developed (Empson et al. 1968, Kogan 1998). These IPM strategies were quickly incorporated into most industries, including the arboricultural industry, by the 1970s (Hock 1984, Holmes and Davidson 1984, Wiseman and Raupp 2016). Today, IPM is the preferred approach for insect and arthropod management within the industry, according to the arboriculture industry’s best management practices for plant health care (Wiseman and Raupp 2016).

Managed residential and commercial landscapes (MRCLs) typically, but not exclusively, exist in urbanized areas with varying levels of infrastructure and impervious surfaces. These landscapes can include park spaces, yards, gardens, residential homes, or commercial sites (e.g. warehouses, office buildings, university, corporate, or government campuses), street and parkway trees, or any other space that is actively managed primarily for aesthetic and/or social benefits (e.g. shade, flood control, tourism). Additionally, while we focus on woody ornamental and shade plants, it is important to acknowledge the abundance and significance of turf grass and herbaceous annual and perennial plants in these landscapes. While commonalities exist between these spaces and other managed systems (e.g. agricultural crop systems, especially those containing tree crops for timber or fruit production), MRCLs pose unique challenges for the care of woody plants.

The MRCL environment has impacts on plant health that can differ from other managed systems, with the magnitude of the impacts varying widely depending on site characteristics (e.g. a downtown core vs. a public park vs. a residential property) (Carol-Aristizabal et al. 2024, Moore et al. 2025, Vuono et al. 2026). Woody plants are often subjected to stressful conditions, which compromise plant resist and/or tolerance to biotic stresses (e.g. Verdugo et al. 2015, Showalter, Villari et al. 2018). This impeded “bottom-up” control of insect pests can have serious consequences for the health of the plant as well as population dynamics of the pest (see Showalter, Raffa et al. 2018). With this, top-down control by natural enemies (e.g. insect predators and parasitoids) is also influenced by the urban environment. Habitat and topsoil loss (Edman et al. 2004, Pavao-Zuckerman and Coleman 2007, Burkman and Gardiner 2014, Korányi et al. 2022), the over-use of insecticides (Duchet et al. 2018), habitat fragmentation and isolation, and host plant heterogeneity and density can all influence insect population dynamics (Held 2020) and herbivory levels (Nuckols and Connor 1995, Kozlov et al. 2017, Quiroga et al. 2026).

Despite these conditions, these landscapes can be designed and maintained in a way that minimizes the deleterious effects of urban environments, part of the foundation of the IPM approach (Wiseman and Raupp 2016). Diversification of plant material enhances top-down control of pests (Shrewsbury and Raupp 2006, Raupp et al. 2010, Frank 2014) and may reduce the need for management interventions such as pesticide applications (Riley et al. 2022). Bottom-up management of insect pests can be improved through the selection and use of resistant plant varieties (e.g. Johnson et al. 2001, Miller and Rigsby 2024) and reducing plant stress (Frank et al. 2013). Despite these strategies, pest management remains a fundamental issue in the management of these spaces. However, the semiochemical mediation of plant, pest, and natural enemy interactions represents an opportunity to develop new approaches and strategies in the IPM of MRCLs.

### Chemical Ecology in the IPM of Managed Residential and Commercial Landscapes

Ecology seeks to understand the relationships between organisms and their interactions with the physical environment (Friederichs 1958), and the sub-discipline of chemical ecology seeks more specifically to elucidate the role that natural chemicals play in mediating these interactions (Hartmann 2008, Raguso et al. 2015). The substances that drive behavioral or physiological responses between individuals of the same or separate species are referred to as semiochemicals (Law and Regnier 1971), which have critical roles in facilitating plant–insect interactions (Beck et al. 2017, Serdo 2024). The leveraging of these semiochemical-mediated interactions for the purpose of mitigating damage to plants by insect pests could be considered the raison d’être of the discipline (Raguso et al. 2015).

Semiochemical-based pest management fits within the framework of IPM incredibly well, and there are several successes utilizing these approaches in crop systems, including in forestry timber production (e.g. Ridgway et al. 1990). However, the number of published studies demonstrating their effectiveness in MRCLs is lacking. Here, we offer our experience and perspective on the use and future adoption of these approaches for the arboriculture industry. We will discuss select instances of our evaluation of existing products on the market in the United States, Canada, and the United Kingdom, benchmarks of success and the likely requirements for the widespread adoption of semiochemical-based approaches in industries that manage woody plants in MRCLs, and possible future research and development directions.

## Semiochemical-Based Management Approaches for Woody Plant Pests

Semiochemical-based pest management strategies have been used to manage woody plant pests for many years, primarily in the context of natural forest or commercial crop systems such as forestry plots and fruit and nut orchards. A prime example is the use of mating disruption to manage spongy moth (*Lymantria dispar* L.) (Lepidoptera: Erebidae) using *cis*-7,8-epoxy-2-methyloctadecane, a relatively cost-effective and ecologically benign strategy used in North American, Japanese, and European forests (Price et al. 2011, Minegishi et al. 2024, Bohinc et al. 2026). Mating disruption of spongy moth is a critical part of the US Forest Service’s Slow the Spread program (Coleman et al. 2023) that reduces populations below Allee thresholds (Thorpe et al. 2007, Tobin et al. 2007, Walter and Liebhold 2023).

The management of bark beetles in North American forests with semiochemical-based techniques also has a successful history (Lindgren and Miller 2002, Seybold et al. 2018, Afzal et al. 2023, Sullivan 2024). The terpenoid ketone, verbenone, can reduce host attraction of at least 38 species of bark beetles (Frühbrodt et al. 2024), including particularly devastating outbreak species such as mountain pine beetle (*Dendroctonus ponderosae* Hopkins) (Coleoptera: Curculionidae) (Progar 2003), red turpentine beetle (*Dendroctonus valens* LeConte) (Coleoptera: Curculionidae), and western pine beetle (*Dendroctonus brevicomis* LeConte) (Coleoptera: Curculionidae) (Fettig et al. 2009). Additionally, the compound 3-methyl-2-cyclohexenone (MCH), an anti-aggregation pheromone for Douglas-fir beetle (*Dendroctonus pseudotsugae* Hopkins) (Coleoptera: Curculionidae), has been commercially available since the 1970s and is arguably the most effective anti-aggregation pheromone used for the management of any bark beetle species (Fettig et al. 2025).

In fruit and nut orchards, semiochemical-based techniques, usually in conjunction with biological control, are also relatively well established for many of the most damaging pests, such as codling moth (C*ydia pomonella* L.) (Lepidoptera: Tortricidae), light brown apple moth (*Epiphyas postvittana* Walker) (Lepidoptera: Tortricidae), and oriental fruit moth (*Grapholita molesta* Busck) (Lepidoptera: Tortricidae) (Ioriatti and Lucchi 2016, Pålsson et al. 2022). Strategies have been successfully developed, leading to widespread adoption by growers (Porcel et al. 2015), and the economic injury thresholds for most pests are well understood, with decisions made based on pheromone trapping (Suckling 2000). However, relative to natural systems and forestry plantations, fewer products may be labeled for edible species, and pre-harvest intervals may restrict application timing. Therefore, semiochemical-based management of tree fruit and nut pests could be considered relatively less established.

Very few semiochemical products are labeled and commercially available for the management of common pests of woody ornamental and shade plants grown in MRCLs. The products that do exist are usually designed for trapping and monitoring particularly notable and damaging pests or were intended for use in natural or agricultural environments. For example, Japanese beetle (*Popillia japonica* Newman) (Coleoptera: Scarabaeidae) trapping has occurred for decades and utilizes a blend of host kairomones (phenethyl propionate, eugenol, and geraniol) coupled with (*R, Z*)-5-(1-decenyl)dihydro-2(3H)-furanone, a synthetic female sex pheromone (Guignard et al. 2025). It was likely a benefit to the arboriculture industry that Japanese beetle is also a major pest of turf grass (Guignard et al. 2025), as this likely promoted investment into management, further illustrating that the methods used in managing woody plants on MRCLs are rarely developed for these specific situations, exclusively.

Various commercial pheromone-based monitoring traps for clearwing borers, such as the dogwood borer (*Synanthedon scitula* Harris) (Lepidoptera: Sesiidae), are readily available, but again, these products were usually developed with crop systems in mind, as is the case with the management of dogwood borer in apple (*Malus* spp.) orchards (Zhang et al. 2013). Although clearwing borer traps and lures can be used in MRCLs, Braxton and Raupp (1995) note several prominent concerns, including frequent misidentification of captured species by practitioners, unclear relationships between trap catches and field populations, and lack of clarity regarding the range of borer species that are attracted to a single pheromone lure. These are issues that we argue still exist over 30 years later and represent a barrier to widespread adoption.

## Trialing Experiences with Semiochemical Approaches

Contemporary research in semiochemical-based management approaches in MRCLs is a mix of formal published research by professional scientists (e.g. Siekmann et al. 2009) and various industry or government professionals (including scientists and practitioners) trialing strategies on their own. Information from these informal trials may be difficult to interpret due to low replication, inconsistent experimental or environmental conditions, or the lack of quantitative responses. For example, verbenone appeared to successfully protect municipal pines (*Pinus* spp.) in Colorado, United States, at the urban–forest interface from mountain pine beetle (Bowser et al. 2026). These factors do not inherently make these trials uninformative, but it does complicate interpretation.

### Box Tree Moth Mating Disruption

A historically important estate in Buckinghamshire County, United Kingdom, contained a formal Victorian garden bordered with boxwood (*Buxus* spp.) plants that were heavily infested with box tree moth (BTM; *Cydalima perspectalis* Walker) (Lepidoptera: Crambidae). This insect is native to East Asia but has established and caused defoliation and mortality of boxwood across its invaded range of Europe, the Middle East, and North America (Armanda 2025). Due to the severe damage caused by BTM and the number of pesticide applications that would be required to aesthetically maintain these hedges, management at the estate decided to remove the entire boxwood planting. Prior to the scheduled removal of the plants, we were able to convince the property management team to allow us to trial a BTM mating disruption product: Box T Pro Press (M2i Life Sciences, Parnac, France) containing (*Z*)-11-hexadecenal BTM sex pheromone.

**Fig. 1.**
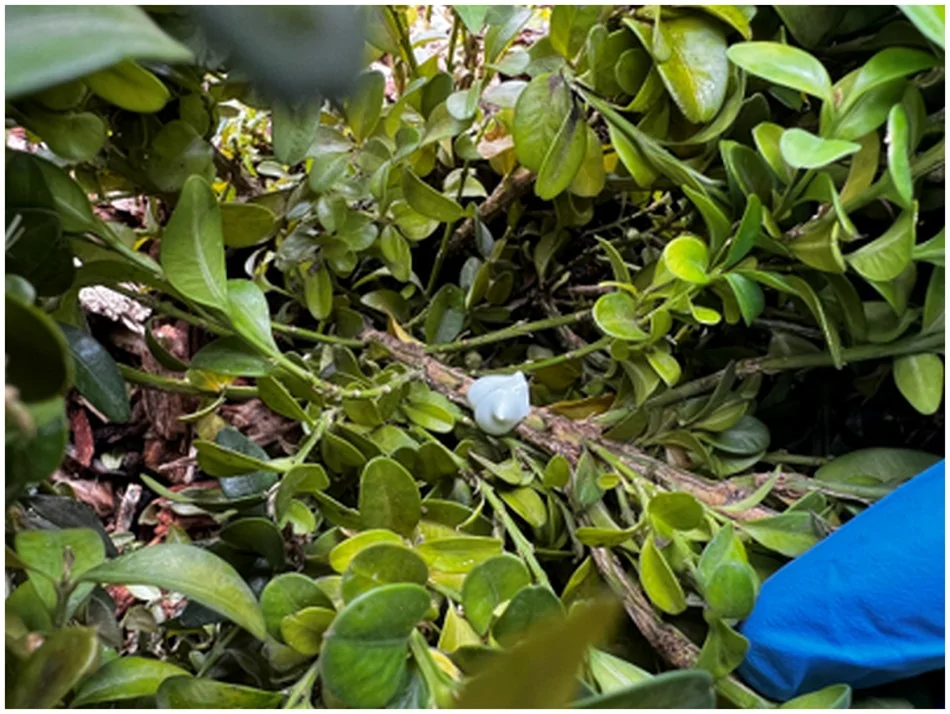
Viscous blob of (*Z* )-11-hexadecenal *Cydalima perspectalis* anti-aggregation sex pheromone being applied within a boxwood hedge in 2024. Image credit: Kevin D. Chase.

Prior to Box T Pro Press application, 2 spot treatments of pyrethrum, timed 7 days apart, were made to reduce the active caterpillar population, and 1 fertilizer application was made to encourage new growth on plants. The site managers conducted the pyrethrum and fertilizer treatments, and no information on rates or products was made available. The first Box T Pro Press application occurred on 22 June 2023 when male moths were active (confirmed via pheromone trapping locally at the site). Viscous blobs of Box T Pro Press were applied at the union of 2 stems with a caulk gun (Fig. 1) and spaced 2 m apart along the hedgerow. The total number of blobs applied to the hedgerow was not counted. Caterpillar activity was then monitored by the estate property management team, who reported no evidence of caterpillars within the planting, despite high numbers of caterpillars observed on neighboring properties. A second application of Box T Pro Press was applied on 13 May 2024, and very few caterpillars were observed within the planting during this application and for the rest of the 2024 growing season. Local trapping across Berkshire County, United Kingdom (adjacent to Buckinghamshire County), confirmed that *C. perspectalis* male adults were lower in 2024 than in 2023 (Chase et al. 2025). This level of BTM management resulted in the recovery of boxwood plants at the site and the ultimate decision to preserve the boxwood hedge by estate managers.

This trial agrees with field observations by other industry professionals in Europe (personal communication—Chris Poole, European Box and Topiary Society) on the high efficacy of Box T Pro Press in protecting boxwood hedges from BTM defoliation. Unfortunately, no (*Z*)-11-hexadecenal product is commercially available in North America as of the publication of this paper, despite this pest rapidly spreading across the continent (Armanda 2025). Boxwood tends to be a highly valued ornamental plant on residential and commercial properties, estates, and public gardens, and many homeowners and managers will invest heavily in their maintenance with little tolerance for damaged, declining, or dead plants. In our experience, without a (*Z*)-11-hexadecenal option, managers are forced to apply multiple applications of insecticides to boxwood plantings over the growing season due to this pest’s overlapping generations throughout a multivoltine lifecycle.

### Douglas-Fir Beetle Anti-Aggregation Pheromones

In 2019, a localized outbreak of Douglas-fir beetle was observed killing Douglas-fir trees in small forest patches and across the urban landscape in Spokane, Washington, United States. We worked with a homeowner to manage the several mature Douglas-fir trees on their ∼1 ha plot using a combined chemical and semiochemical approach. We applied 2 bark applications of a bifenthrin product (2.5 ml/l water; Baseline; FMC Corporation, Philadelphia, Pennsylvania, United States) to the bole of all Douglas-fir (*Pseudotsuga menziesii* [Mirbel] Franco), timed at roughly a 6-week interval, and placed MCH anti-aggregation pheromone lures on all trees starting on 5 April 2019. Two Lindgren funnel traps paired with lures containing 3-methyl-2-cyclohexenone, a commercial Douglas-fir kairomone mix, low-release ethanol, and frontalin (Synergy Semiochemicals, Vancouver, British Columbia, Canada) were both suspended between 2 trees using a rope. Within a few weeks, a spillover event occurred when beetles began attacking trees that were used to anchor the funnel traps, and we therefore moved the traps to non-host deciduous trees as far away from host trees as possible. After this, no further damage was observed on trees that were targeted for protection. This management approach continued uninterrupted through 2025, with no further observations of beetles attacking protected trees at the property, despite many neighboring Douglas-fir trees succumbing to beetle attack.

We were highly aware and concerned about the possibility of “pushing” beetles to neighboring properties, and about the property owner being responsible for tree mortality as a result. However, Ross et al. (2015) found no evidence that tree mortality is higher on adjacent properties where MCH is being utilized, and we concluded that the risk level to trees on adjacent properties was minimal enough to conduct the trial. Our observations support this conclusion, as there appeared to be no difference between Douglas-fir tree mortality rates on adjacent properties and properties elsewhere in the area, and these decline rates appeared temporally consistent based on visual inspections by the local arborists in Spokane, Washington (authors’ personal communication). Based on this experience, we recommend plant health care managers incorporate MCH anti-aggregation lures into an IPM strategy to protect host trees from Douglas-fir beetle attack in the urban landscape.

### Black Turpentine Beetle Overspill on Martha’s Vineyard, Massachusetts, United States

In 2019, a project was initiated to determine if the monoterpenoid cineole (a.k.a., eucalyptol) could repel black turpentine beetle (*Dendroctonus terebrans* Olivier) (Coleoptera: Curculionidae) in eastern Massachusetts, United States. Localized black turpentine beetle outbreaks are common on pitch pines (*Pinus rigida* Mill.) in residential landscapes in this region (authors’ personal observation). Four 12-funnel Lindgren funnel traps were installed and paired with one of the following semiochemical lure combinations: (i) Ethanol, α-pinene, and β-pinene; (ii) Lure 1 with endo-brevicomin (an aggregation pheromone component); (iii) Lure 1 with frontalin (an aggregation pheromone component); or (iv) Lure 1 with cineol. The 4 trap and lure combinations were installed at 5 sites—2 sites on the island of Martha’s Vineyard and 3 sites across the Cape Cod, Massachusetts peninsula. Traps with lures were placed at least 5 m from each other, were attached to a rope using a carabiner, and the rope was tied to 2 pitch pine trees. Traps were installed between 29 April and 3 May 2019 on private properties that granted permission for this trial. All lures and traps were provided by Synergy Semiochemicals (Vancouver, British Columbia, Canada).

**Fig. 2.**
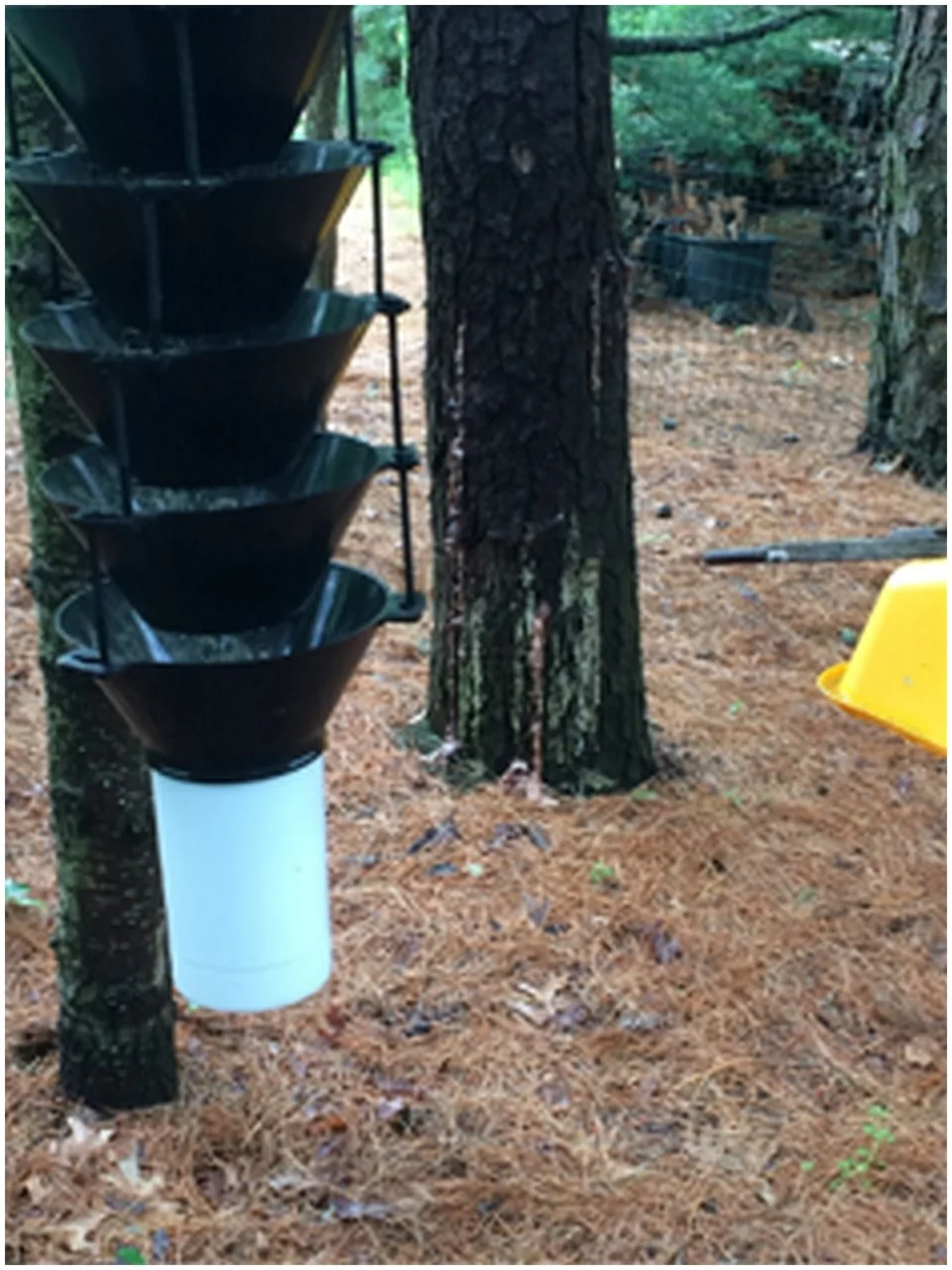
Black turpentine beetle overspill attack on pitch pine in 2019 on Martha’s Vineyard, Massachusetts, United States. Image Credit: Kevin D. Chase.

On 26 June 2019, we were notified that several sites were experiencing black turpentine beetle mass attack on trees that were used to anchor traps (Fig. 2). This experience led to all involved stakeholders requesting disengagement from the study and resulted in a general apprehension of any semiochemical-based strategy from local arborists. This experience illustrates the difficulties presented by working with the property owners and managers, and even industry personnel such as arborists, in conducting this type of research. This also demonstrates that first-hand, negative experiences via involvement in research can profoundly change personal attitudes toward a strategy, as it may be difficult for these property owners and arborists to be willing to utilize these strategies in the future.

### Japanese Beetle Feeding on Linden Trees Manipulated by Pheromone Lures

**Fig. 3.**
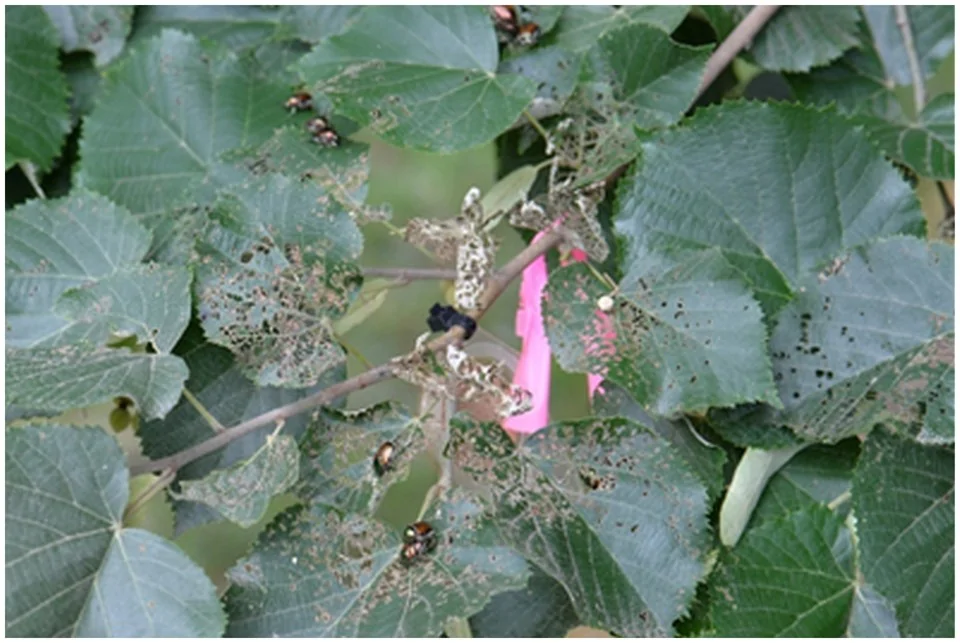
Japanese beetle congregation and feeding damage in close proximity to a pheromone lure on a linden tree in Charlotte, North Carolina, United States. Image credit: Kevin D. Chase.

In 2021, we conducted a study at The R.A. Bartlett Tree Research Laboratories and Arboretum in Charlotte, North Carolina, United States, assessing the efficacy of systemic insecticides against Japanese beetle (*P. japonica* Newman) (Coleoptera: Scarabaeidae) feeding on linden (*Tilia* spp.) trees (Littlejohn et al. 2022). Lures containing female sex pheromones were used to attract Japanese beetles to trees in a “lure and kill” fashion (see El-Sayed et al. 2009, Gregg et al. 2018). The goal of this study was to assess the efficacy of conventional insecticides, not necessarily to address questions regarding semiochemical management. However, the localized feeding damage occurring around lures that were placed directly on foliage was an interesting observation. Instead of drawing in beetles that then attacked the whole tree, as expected, feeding damage was almost exclusively detected on foliage near lures (Fig. 3). Though not yet explored, this observation of the spatial manipulation of beetle feeding by pheromone lures highlights the potential to reduce conventional insecticide use by drawing beetles to a specific part of the crown and applying product to that site, similar to the function of attract-and-kill systems. If effective, this strategy would extend benefits to pesticide applicators, such as drawing beetles to lower and/or less visible portions of the canopy or even to adjacent shrubs, where applications are made easier and concerns regarding drift management and crown coverage are reduced.

### Bagworms on Bald Cypress

In the late 1990s, an isolated outbreak of bagworms (*Thyridopteryx ephemeraeformis* Haworth) (Lepidoptera: Psychidae) was damaging a group of bald cypress (*Taxodium distichum* (L.) Rich.) in downtown Charlotte, North Carolina, United States. Trees were situated such that no pesticide applications could be made, given drift and collateral spray concerns. An Entomologist with The R.A. Bartlett Tree Research Laboratories and Arboretum, Donald Booth (ret.), deployed bagworm female sex pheromone lures, 1-methylbutyl decanoate, across the site, which has been shown to have mating disruption properties when dispensed at high rates (Leonhardt et al. 1983, Klun et al. 1986). The 1-methylbutyl decanoate was provided by staff with the Department of Entomology at Cornell University. Within 1 growing season, continuous observation of the site showed that the bagworm population had been reduced to the point to which individuals were barely detectable and almost no damage was occurring to the trees. It was assumed that the population reduction was likely attributable to the placement of the pheromone lures due to the isolation of this specific bagworm population, as adult females are immobile and never leave their silk bags, and conventional pesticides were not used. We believe these assumptions to be reasonable. It is apparent to us that *T. ephemeraeformis* (and likely other bagworm species) is a prime candidate for semiochemical use in ornamental landscapes due to the combination of females being immobile, the possibility of seasonal mis-matches between male and female emergence (Rhainds 2026), the common practice of planting evergreen hosts as continuous “green screens,” and the availability of a strong sex pheromone that can disorient males when dispensed in high amounts.

### Natural Enemy Attraction and Biological Control through Methyl Salicylate Lures

While most of our semiochemical-based research efforts have focused on herbivore biology, ecology, or management, we have also explored strategies centered around the manipulation of natural enemies with the goal of enhancing biological control services. Many arthropod natural enemies utilize the chemical signals given off by plants under attack by herbivores to orient to a prey resource (Rodriguez-Saona et al. 2011). Methyl salicylate (MeSA) is a widely studied herbivore-induced plant volatile that influences the recruitment and behavior of predatory arthropods in agricultural contexts (Lee 2010, Mallinger et al. 2011, Salamanca et al. 2019). It is often commercially available for pest management applications in ready-to-use, controlled-release packets (e.g. “PredaLure,” purchased from AgBio Inc., Westminster, Colorado, United States) and marketed as a pesticide-free, non-toxic means of suppressing crop and garden pests (http://www.agbio-inc.com/predalure.html). Such a product is inherently appealing to both pest management professionals and members of the public who are interested in either reduced-risk or low-input treatment approaches in MRCLs.

**Fig. 4.**
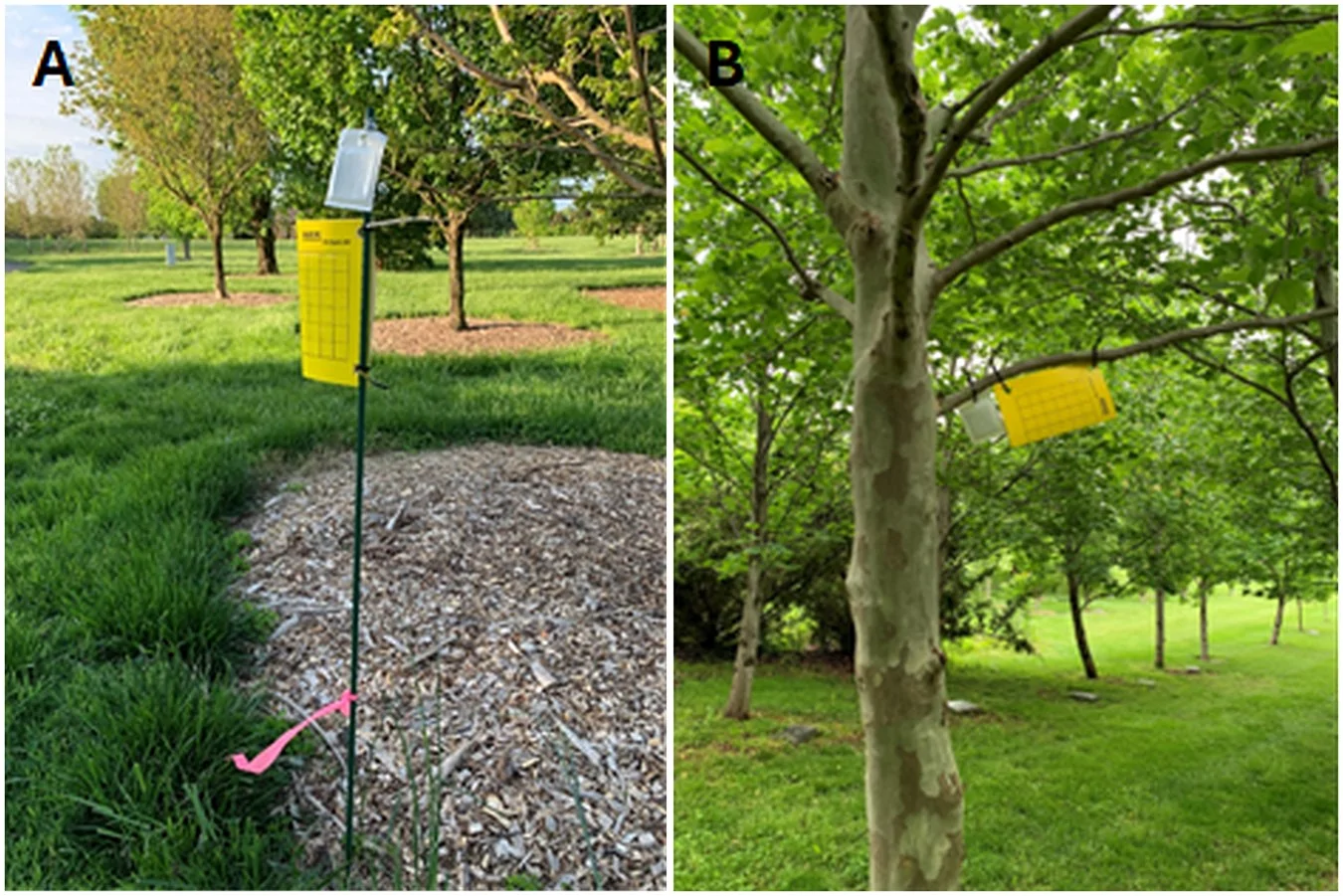
A) PredaLure packet (white) with yellow sticky trap affixed to a stake in 2020 at The R.A. Bartlett Tree Research Laboratories and Arboretum in Charlotte, North Carolina, United States. B) PredaLure packet and yellow sticky trap attached to a study tree in 2022 at the site in Alexandria, Virginia, United States. Image credits: Christopher B. Riley.

From 2020 to 2022, we conducted several preliminary trials examining the effects of MeSA packets (i.e. PredaLure) on natural enemy collections, pest populations, and pest damage. At our laboratory in Charlotte, North Carolina, in 2020, 4 double-sided yellow sticky traps were deployed in square configurations with 50 m edges on stakes 1.5 m tall across 8 different collections (*n* = 32) of the arboretum (Fig. 4A). Half of all stakes (diagonal pairs of the squares; *n* = 16) had a PredaLure packet attached adjacent to the sticky trap. Traps were left for a week at a time and replaced over the growing season. In general, traps with lures caught a greater abundance of key natural enemy taxa, including members of Dolichipodidae, Syrphidae, Anthocoridae, and Hymenoptera.

Another preliminary trial was conducted over the 2021 growing season at our laboratory location to examine changes in azalea lace bug (*Stephanitis pyrioides* Scott) (Hemiptera: Tingidae) abundance and stippling damage as a function of MeSA lure usage. Twenty-seven 3-gallon azaleas (*Rhododendron* spp.) were purchased from a local garden center and were staked in a graveled plant holding yard in such a way that there were 3 individual plants spaced out evenly within a row (∼12 m apart) and 9 rows in total (∼3 m apart). At random, 1 of each of the 3 plants within a row was assigned to the untreated control group, a group receiving a PredaLure packet, or a netted exclusion group (to prevent arthropod access throughout the growing season). At the onset of the study, 3 adult lacebugs were added to the crown of each plant, and counts were performed on the total number of lacebugs and level of stippling damage. By the end of the growing season, azaleas with PredaLure packets generally had fewer lacebugs and lower levels of stippling damage than those in the other treatments.

Finally, a trial was conducted in 2022, like the original 2020 trial, at a historical property in Alexandria, Virginia, United States. Compared to the 2020 trial location (Charlotte, North Carolina), this site was embedded in a more urbanized landscape. For this trial, traps with (*n* = 25) and without (*n* = 25) lures were deployed across established same-species tree plantings (e.g. rows of *Acer rubrum* L., clusters of *Lagerstroemia indica* L.). Study trees, and therefore traps and lures, were generally much closer to one another than the 50 m spacing used at our laboratory (Fig. 4B). By the end of the season, traps from this trial had caught far fewer arthropods than had been collected in Charlotte, and there were few patterns in which taxa were trapped. This aspect of our natural enemy survey trial reinforced that even if MeSA lures have the potential to attract select taxa, other ecological filters of MRCLs on arthropod communities could limit their efficacy, such as those discussed earlier (e.g. patch size and connectivity).

As a research group, we have refrained from making formal recommendations or providing guidance to the industry regarding the utilization of these commercially available MeSA lures, as we feel that there is significantly more research to be done. Theoretically, their use to attract natural enemies of herbivorous insect pests is logical and has been demonstrated in agricultural systems (Lee 2010, Mallinger et al. 2011, Salamanca et al. 2019), but traits unique to MRCLs likely make their successful utilization more nuanced. We foresee the greatest likelihood of success using MeSA lures is in large public gardens and campuses with diverse plantings that host a greater array of natural enemies.

## Challenges to Implementation

When considering the barriers to wider implementation to pest management in MRCLs, we argue that the most critical obstacles to implementing effective semiochemical-based approaches arise from 3 primary challenges. First, “spatial complexity” or the existence of ecologically arbitrary but legally meaningful property boundaries that limit the scale and/or scope of their implementation. Second, “specificity and efficacy” or the subjective nature of success and the gap between statistically significant reductions in pest density and the perceived aesthetic improvements these approaches provide, specifically compared to the use of traditional pesticides. And third, “manufacturing, regulatory, and public perception challenges” or the lack of consistent regulatory frameworks coupled with the lack of willingness of manufacturers to research, develop, and bring products to market and the ever-necessary need for public education.

### Problem Area One: Spatial Complexity

One of the defining features of MRCLs is the proximity of landscapes to each other as well as the legal boundaries that separate and define them. This poses an enormous challenge to the implementation of semiochemical technologies, as presently there can be no assurances or solid expectations regarding outcomes. For example, no guarantees can be made that the alteration of pest dispersal via the use of a repellent will not deleteriously impact plants on an adjacent landscape. Pest thresholds in urban spaces are often based on aesthetics and nuisance tolerance, but some pest attacks (e.g. bark and ambrosia beetles) can result in decline or mortality. While these pests may already be present and attacks could occur in the absence of interventions, there may be some legal liabilities associated with modifying pest behavior and attack dynamics. We are not lawyers and have no formal legal training, but we are not aware of any case law exploring these issues. Industry professionals, like arborists, are not going to take chances that could become legally problematic, and studies finding no evidence of an increased risk of spillover attack (e.g. Ross et al. 2015) are unlikely to provide adequate legal protection.

These boundary issues also result in the inconsistency of semiochemical usage among adjacent properties, posing problems for efficacy. For example, it is well-established that for Lepidopteran mating disruption, pest population density can be critical to the success of these strategies (Cardé and Minks 1995, Witzgall et al. 2008, Frank et al. 2020), with the likelihood of success being greatest when pest densities are low and large areas can be under the influence of pheromones (Frank et al. 2020). Unless arborists or homeowners engage with local municipalities or homeowner associations to implement these strategies at scales larger than residential properties, the likelihood of successful management on one or a few landscapes decreases dramatically.

Alternatively, the lower abundance of individual host plants, relative to crop systems, may result in isolated pest populations that could be highly sensitive to attractive host or conspecific volatiles. Fewer susceptible hosts may naturally result in pest aggregations and therefore improve chances of conspecific encounters in the absence of semiochemical tools. Further, in areas where fewer hosts are present to support a large pest population, there may be localized extirpation of the pest species, which could affect dispersal dynamics among managed landscapes. These irregularly distributed populations among residential properties and communities may then be very susceptible to trapping approaches even if mating disruption tools are less effective. For example, the greatest 2-week capture (22,697 beetles) of pine engraver (*Ips pini* Say) (Coleoptera: Curculionidae) in a 2-year trapping study across the Midwestern United States occurred from a Lindgren funnel trap hung in a crabapple tree adjacent to a corn field where the closest host resource (3 *Pinus resinosa* Sol. Ex Aiton) was approximately 2 km away (authors’ unpublished data). Only when populations are more isolated does this approach show greater promise alone or possibly in combination with other IPM tactics (Wawrzynski and Ascerno 1998).

### Problem Area 2: Product Efficacy and Specificity

In MRCLs, pest management interventions often rely on conventional synthetic insecticides to suppress or eradicate pest populations based on the client’s aesthetic threshold. Alternative management approaches (e.g. cultural, mechanical, biological) can be highly effective and are heavily promoted by industry researchers and educators. However, we find that insecticides are heavily relied on more than other IPM strategies because arborists can easily train and certify applicators, clearly communicate expected outcomes to clients, are able to safely store these products for long periods, and can be consistently relied on to provide satisfactory results that are visually verifiable and even quantifiable. For semiochemical-based approaches to become more widely adopted, they will need to fit more closely into this general industry model, particularly regarding having similar management efficacy relative to conventional insecticides.

Any developed products or strategies that do not have similar efficacy to conventional insecticides are unlikely to be commercially sustainable. For example, permethrin is a common insecticide used to reduce attack by bark and ambrosia beetles on woody plants, and it was found to outperform verbenone and MeSA repellents in the prevention of ambrosia beetle infestation of red maple bolts (Govindaraju and Joseph 2025). Reduced efficacy or inconsistencies over time when using these products may generate more apprehension when considering pests that contribute to host mortality relative to those that may not consistently warrant management.

Many semiochemicals are highly specific to the target species with regard to behavioral effects (e.g. certain sex pheromones), but other volatiles exhibit more generalized responses among other insects and plants. For example, MeSA serves as both a stress-induced volatile emitted by hosts that attract pests, such as the Asian citrus psyllid (*Diaphorina citri* Kuwayama) (Hemiptera: Psyllidae) (Martini et al. 2018), but also can be an attractant to many beneficial insect species, as discussed previously. How these signals are perceived among different insect pests will dictate how broadly they can be used in variable landscapes. An arborist can be very confident that an application of permethrin to manage bark and ambrosia beetles will almost certainly not result in a lacebug outbreak on untreated plants, and the arborist requires this same level of confidence, provided by repeated studies and published research when deploying, for example, a MeSA lure.

A greater understanding of pheromone lure release rates and dosages on the response of the target pest is needed (El-Sayed et al. 2009). It is known that pheromone responses of bark beetles to the same compound shifts from repellent to attractant as the dosage decreases. For example, the repellency or attraction of *Ips latidens* (Leconte) (Coleoptera: Curculionidae), pine engraver, and mountain pine beetle to verbenone was based on the dose-dependent response of these beetles to lures placed in a *Pinus contorta* Douglas forest in British Columbia, Canada (Miller et al. 1995). While pheromone lure dispensers are designed to release a steady rate of a specific concentration to induce the appropriate behavioral response, it is possible that lures left in landscapes for periods exceeding these temporal thresholds could become problematic. Therefore, more work needs to be conducted to better understand how long verbenone lures should be left in landscapes before the dosage released by this compound possibly shifts from a repellent to an attractant.

### Problem Area 3: Manufacturing, Regulatory, and Public Perception

Another issue arises from the often-high specificity of semiochemicals to the target pests and their effects on behavior, which requires a significant amount of research into the basic biology and chemical ecology of not only individual species but also closely related heterospecifics. This is a significant advantage of conventional insecticide development over semiochemical-based strategies. Furthermore, the high costs associated with product development, registration, and marketing likely means that semiochemical tools may never be developed for less serious pests or those that are already easily managed with existing tools. Bakthatvatsalam et al. (2022) suggest that the development of cost-effective semiochemical formulations and more effective dispensers could allow for more widespread utilization of these technologies. We agree with this assessment, specifically in the case of existing and available technologies for use in MRCLs.

Additionally, the regulatory framework for traditional pesticide registration in the United States and many other countries is well developed, and companies developing or seeking to register new products are usually well aware of the processes. However, the procedures can be substantially less clear for registering new semiochemical, biopesticide, or microbial products (Weatherston and Minks 1995). There is also a lack of consistent or complementary regulatory frameworks among countries, and this can impact the willingness of a company to develop such products if the market is seen as restricted. Yet, even when markets are not restricted, there may still be development hesitancy associated with the perceived ability to sell a product based on pest severity or host density.

Lastly, public perception and demand will have a significant impact on the future development and use of these approaches. There is a general trend toward pesticide hesitancy among consumers, which may drive up demand for alternatives; however, in some cases, there has been public outcry when semiochemical tools have been deployed at scale by regulatory authorities. For instance, when California Department of Food and Agriculture agents proposed aerial spray programs targeting light brown apple moth, there was significant public outcry that led to the program being rescinded shortly after sprays were initiated (Carey et al. 2023). These same social dynamics are almost certain to occur if, for example, a semiochemical-based strategy was to be deployed at the scale of a neighborhood or municipality.

## Potentially Productive Future Research Avenues

Not all challenges to widespread implementation of semiochemical-based pest management strategies are ameliorable with deliberate and focused research and development activities, such as regulatory challenges. However, targeted research and development does have the potential to lead to more widespread adoption in MRCLs either by leading directly to new plants and products for utilization or simply by informing industry personnel and property owners and managers of expectations. Here, we suggest some potentially productive research and development efforts that could lead to greater widespread adoption of semiochemical-based pest management methods.

### The Development of New Woody Plant Cultivars and Varieties

Many researchers involved with the management of exotic, invasive, and destructive forest pests, such as emerald ash borer (*Agrilus planipennis* Fairmaire) (Coleoptera: Buprestidae) in North America, have argued for a renewed approach to the management of these pests (e.g. Bonello et al. 2020). Specifically, the incorporation of and investment in resistance development (Showalter, Raffa et al. 2018). Horticulturalists and woody plant breeders certainly have resistance at the top of mind, as cultivars and varieties are routinely produced with elevated levels of resistance to major pests. For example, the US National Arboretum introduced *Tsuga chinensis* × *Tsuga caroliniana* “Traveler” and *T. chinensis* × *T. caroliniana* “Crossroads” relatively recently, both resistant to hemlock woolly adelgid (*Adelges tsugae* Annand) (Hemiptera: Adelgidae).

However, there are 2 forms of plant resistance to herbivores: antibiotic resistance, characterized by direct and deleterious effects on the insect, and antixenotic resistance, which is characterized by non-preference (e.g. Rigsby et al. 2014). Traditional breeding with antixenosis in mind as well as antibiosis would be a simple and straightforward approach to incorporating semiochemical-based management onto MRCLs. For example, transgenic kairomone manipulation has been shown to successfully reduce pest attraction (e.g. Salvagnin et al. 2018) and enhance repellency (e.g. Alquézar et al. 2021). Alternatively, the increase of emission rates of known natural enemy attractants to increase their attraction could enhance top-down control of pests (Beale et al. 2006, Li et al. 2024).

Questions remain regarding the field efficacy of plants with enhanced antixenosis or natural enemy attraction, even in agricultural settings. For example, Bruce et al. (2015) field tested transgenic wheat (*Triticum aestivum* L.) with higher emission rates of an attractant to aphid parasitoids to assess any enhanced parasitism rates associated with the transgenic plants, but enhanced levels of aphid parasitism were not detected after 2 years. Despite these challenges and the investment necessary to develop plants with these traits, we believe this is an important research avenue and could produce practical results.

### Lure Specificity and Design

One area of research and development that continues to require major investment is the selectivity of semiochemical methods, but this can be an enormous research challenge depending on the system. For example, bark beetle pheromone plume profiles can vary regionally, by host, and from beetle-to-beetle (Aukema et al. 2010). Trapping for emerald ash borer using established trap and lure combinations (Crook et al. 2008, Poland et al. 2019) tends to result in a tremendous level of bycatch, often click beetles (Coleoptera: Elateridae), that can confuse monitoring personnel and be difficult to sort through (authors’ personal observations; authors’ personal communication, Frederic Miller, The Morton Arboretum).

This is essentially the issue identified over 30 years ago by Braxton and Raupp (1995). This high degree of non-specificity may be acceptable for trained personnel conducting monitoring activities, but this can become an enormous obstacle to the widespread use of these tools in the management of MRCLs. Japanese beetle trapping is an example of the ideal combination of required efficacy, ease-of-use, and interpretation of trap capture information. While it may be easier to diagnose Japanese beetles relative to bycatch specimens, researchers found that removing the geraniol component of the lure can significantly reduce the number of bees caught in traps without affecting the collection of Japanese beetles (Sipolski et al. 2019). The development and continued improvement of tools with a high degree of specificity for target pests or groups of pests, that minimize bycatch, would encourage their widespread adoption.

Over the last several decades, a trend in semiochemical-based trapping research has been to explore the potential for implementing multiple volatile components into a single lure with the purpose of monitoring a greater range of pest species (Quilici et al. 2012), including combining host kairomones and insect pheromones (Reddy and Guerrero 2004). This approach has the potential to reduce costs without compromising the likelihood of detecting pests (Chase et al. 2018, Fan et al. 2019, Stringer et al. 2019, Hoch et al. 2020). Multi-lure approaches have been investigated in agricultural settings (e.g. Siderhurst and Jang 2010, Lasa et al. 2015, Baroffio et al. 2018, Giri 2022, Ricciardi et al. 2026). However, substantially more research is required to develop workable lures for MRCLs, as not all semiochemical combinations are compatible (Ricciardi et al. 2026).

## Public Response and Industry Logistical Considerations

The decision to use pesticides in IPM programs is often driven by aesthetics, budget, labor required, and environmental impact (Park Brown 2000). Property owners/managers who use pesticides on their landscapes value plant health and aesthetics as well as rapid and effective results, with some doubt that pests can be managed without using pesticides (Park Brown 2000, Kratsch et al. 2017). Those in urban and suburban areas often consider perceptions of their landscape aesthetics by neighbors and the public, many of whom associate attractive landscapes with increased property values (Kratsch et al. 2017). These aesthetically driven clients will require high efficacy from semiochemical products, have low thresholds for visible plant damage, and prefer discreet placement of semiochemical deployment equipment.

Many property owners/managers feel that a combination of methods can be used to monitor and manage pests and that pests should only be managed if they are causing significant damage (Kratsch et al. 2017), and these individuals may be more receptive to incorporating semiochemical products into an IPM program. This view may become more widespread as the public perception of pesticide use is increasingly negative (Jun et al. 2023). Increased semiochemical-based management options could create new client opportunities with those with negative views of traditional pesticides. Understanding the fundamental views of property owners and managers will help identify clients in which semiochemical treatments could be successfully incorporated into pest management programs.

Many property owners/managers will have pre-conceived perceptions of pesticides and IPM practices. Semiochemical-based strategies may be introduced to clients by an arborist, but to do this effectively, the arborist must have a thorough understanding of the use, application, and effectiveness of semiochemical treatments. To inform and encourage clients to adopt a new landscape practice, arborists can provide additional resources such as fact sheets, which The Virginia Polytechnic Institute and State University (Virginia Tech) cooperative extension ranks as “the most effective tool for public education on IPM topics” (Park Brown 2000, Frank and Blevins-Wycoff 2024). Arborists must also be able to correctly price the service to successfully sell it and be profitable, which will require research to understand the time required to apply the treatment, materials needed, the number of times the property needs to be visited, and the overall time period for the desired result to be achieved. Ultimately, this cost needs to be both profitable for the business and reasonable for the result seen on the landscape by the client.

Successful implementation of semiochemical products will require appropriate preparation, planning, and application. Preparation begins with adequate training and identification of semiochemical products for target pests. Reliable sourcing of these products as well as stock availability and quick delivery will enable practitioners to reliably sell and deploy the service. Many semiochemical products require climate-controlled storage. For example, some lures and anti-aggregation products require long-term storage at or under 4 °C and have a shorter shelf life than most traditional pesticides (M2i Biocontrol, Synergy Semiochemicals). Creating and maintaining available space in operating buildings as well as on application vehicles through use of coolers and ice packs before deployment in the field will be necessary to maintain product quality and efficacy. The personal protective equipment (PPE) required for applicators of semiochemical products on the market includes chemical-resistant gloves, eye protection, and, in some cases, a face mask (Chemtica Internacional, M2i Biocontrol). These requirements are at or below the minimum PPE for most traditional pesticides and should be readily available to applicators by employers. Other general tools such as staplers, hammers, and wooden posts may be needed for deployment of semiochemical products and are common in the green industry or easily acquired locally.

Planning of a treatment requires knowledge of pest phenology and the duration of application window relative to the target life stage of the pest. Weather conditions required for successful deployment must be known, for example, Box T Pro Press requires a 24 h rain-free period post application (M2i Biocontrol). Understanding impacts of other treatments on the landscape, such as fungicide applications, is important as typical IPM programs on ornamental landscapes manage various pest and disease issues simultaneously. These factors can be evaluated to plan treatments for maximum efficiency and efficacy.

## Conclusion

In many regions of the world, the regulatory and cultural conditions are beginning to, or already has, placed substantial negative pressure on traditional pesticide use and have limited the development of novel products. This loss of traditional pesticides is forcing researchers to develop alternative approaches to pest management, and the utilization of semiochemical-based strategies is likely going to play a larger role in pest management moving forward. However, several obstacles to the widespread utilization of these strategies exist on MRCLs. For these strategies to become widely adopted, they need to be easy to use, offer the same or similar levels of efficacy that are spatially defined and easy to see (or the changing of client aesthetic thresholds through education), be cost-effective, and be easily and simply communicable to the public and industry personnel. Focused research and development may solve many of these problems, but the circumstances of MRCL spaces, and the unique challenges these environments present, is going to need to be considered by researchers.
